# Time Perception and the Experience of Time When Immersed in an Altered Sensory Environment

**DOI:** 10.3389/fnhum.2017.00487

**Published:** 2017-10-06

**Authors:** Joseph Glicksohn, Aviva Berkovich-Ohana, Federica Mauro, Tal D. Ben-Soussan

**Affiliations:** ^1^Department of Criminology, Bar-Ilan University, Ramat Gan, Israel; ^2^The Leslie and Susan Gonda (Goldschmied) Multidisciplinary Brain Research Center, Bar-Ilan University, Ramat Gan, Israel; ^3^The Edmond J. Safra Brain Research Center for the Study of Learning Disabilities, Faculty of Education, University of Haifa, Haifa, Israel; ^4^Department of Psychology, Sapienza University of Rome, Rome, Italy; ^5^Research Institute for Neuroscience, Education and Didactics, Patrizio Paoletti Foundation for Development and Communication, Assisi, Italy

**Keywords:** time perception, sensory environment, whole-body perceptual deprivation, *Ganzfeld*, time production

## Abstract

The notion that exposure to a monotonous sensory environment could elicit reports indicating aberrant subjective experience and altered time perception is the impetus for the present report. Research has looked at the influence of exposure to such environments on time perception, reporting that the greater the environmental variation, the shorter is the time estimation obtained by the method of production. Most conditions for creating an altered sensory environment, however, have not facilitated an immersive experience, one that directly impacts both time perception and subjective experience. In this study, we invited our participants to enter a whole-body altered sensory environment for a 20-min session, wherein they were asked to relax without falling asleep. The session included white-colored illumination of the chamber with eyes closed (5 min), followed by 10 min of illuminating the room with color, after which a short report of subjective experience was collected using a brief questionnaire; this was followed by an additional 5 min of immersion in white light with closed eyes. The participants were then interviewed regarding their subjective experience, including their experience of time within the chamber. Prior to entering the chamber, the participants completed a time-production (TP) task. One group of participants then repeated the task within the chamber, at the end of the session; a second group of participants repeated the task after exiting the chamber. We shall report on changes in TP, and present data indicating that when produced time is plotted as a function of target duration, using a log–log plot, the major influence of sensory environment is on the intercept of the psychophysical function. We shall further present data indicating that for those participants reporting a marked change in time experience, such as “the sensation of time disappeared,” their TP data could not be linearized using a log–log plot, hence indicating that for these individuals there might be a “break” in the psychophysical function.

## Introduction

Exposure to an altered sensory environment, such as that entailing what Marcusson-Clavertz et al. (2012) have termed a “sensory homogenization procedure,” has a marked impact on subjective experience (Glicksohn, 1991; Wackermann et al., 2008). Sensory homogenization is achieved by means of a *Ganzfeld* (homogeneous perceptual field), which can be experienced if one is placed “…in the midst of an actual fog which would be perfectly evenly illuminated" (Koffka, 1935, p. 111). As Koffka, citing the pivotal experimental study by Metzger, writes, “the observer will ‘feel himself swimming in a mist of light…’.” (Koffka, 1935, p. 111). Koffka (1935, p. 114) expands on Metzger’s procedure for creating such a *Ganzfeld* in the lab: “The observer sat in front of a carefully whitewashed wall…at a distance of 1.25 m. … wings bent towards the observer had to be added on all four sides, care being taken that the inhomogeneities thereby introduced were as small as possible … The illumination was supplied by a projection lantern ….” Ash (1995, p. 230) provides a photograph of this *Ganzfeld* setup.

A more convenient solution to creating a *Ganzfeld* is to employ halved ping-pong balls covering the eyes, coupled with exposure to red-colored stimulation, as pioneered by Hochberg et al. (1951) – this being the technique that we employed in previous research (Glicksohn, 1991, 1992; see also Wackermann et al., 2002, 2008). Yet, as Avant (1965, p. 249) correctly asserted, “It is highly likely that this technique produces a different field from that produced by a larger stimulus field at a greater distance from the eyes.” All such *Ganzfeld* techniques entail pattern reduction, monotony, homogeneity and perceptual deprivation (Suedfeld, 1980, pp. 8–9).

The notion that exposure to a monotonous sensory environment could elicit reports indicating aberrant subjective experience (Marks et al., 1968; Mason and Brady, 2009; Daniel et al., 2014; Daniel and Mason, 2015) presents an interesting inroad into studying the relationship between such aberrant subjective experience and altered time perception. For example, Niedenthal (2002, p. 253) reports that “some visitors to a Turrell Ganzfeld installation at the Stedelijk museum in Amsterdam felt so disembodied they had to crawl through the space on hands and knees … .” And Gadassik (2016, p. 309), reporting on her own experience within a Turrell *Ganzfeld* setup, writes: “My ten-minute Hard program inside Light Reignfall gave me the impression of lasting two minutes, and when my reclining tray was rolled out I worried I had pressed the panic button by mistake. One of the lab coat assistants assured me that almost everyone experiences the Hard program at half the time or less of its measurable duration.”

**FIGURE 1:**
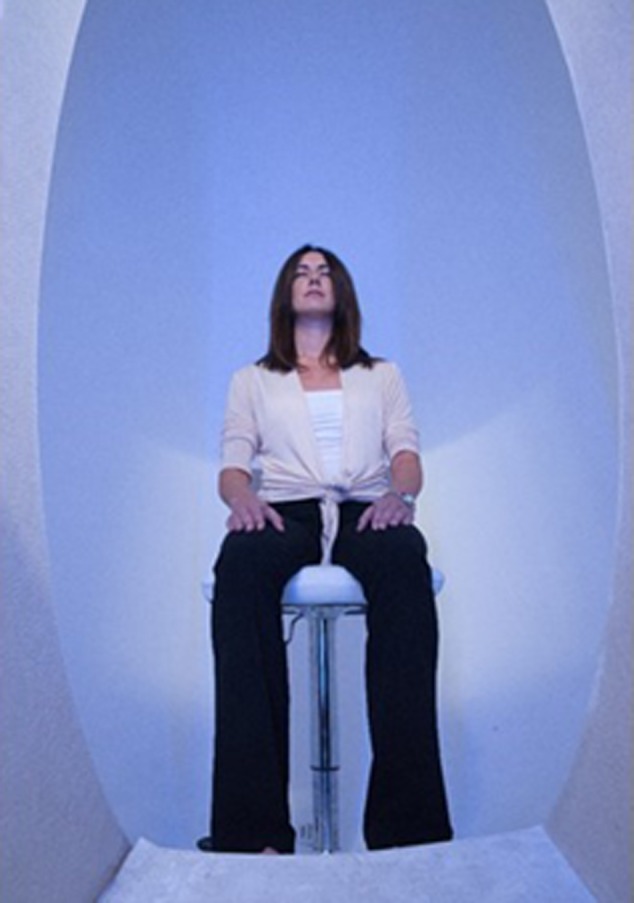
Whole-Body Perceptual Deprivation (WBPD). The person in the photo has volunteered to be photographed for the illustration of the WBPD paradigm, and did not take part in the current research.

Research looking at the influence on time perception of exposure to such altered sensory environments indicates that the greater the environmental variation, the shorter is the time estimation obtained by the method of production; hence, exposure to a monotonous sensory environment should result in a *lengthening* of time production (TP; Glicksohn, 1992, 1996). This is clearly not because such environments are monotonous, hence are inevitably boring, for if this condition were boring, then one would expect a *shortening* of TP (Doob, 1971, p. 292; Zakay, 2014, p. 3). We stress that most conditions for creating an altered sensory environment have not facilitated an *immersive* experience, one that directly affects both time perception and subjective experience (Glicksohn and Berkovich-Ohana, 2012). In the present study, we use a unique environment of Whole-Body Perceptual Deprivation (WBPD; see **Figure 1**) to enable us to investigate the relationship between aberrant subjective experience and time perception.

Our TP task requires the participant to produce a target duration (*P*) by signaling when that duration (*T*) is thought to have elapsed. For example, if the required duration to be estimated is 8 s, individual **A** might produce a duration of 8 s, individual **B** one of 10 s and individual **C** one of 6 s. Note that for all three individuals, produced duration (*P*) is subjectively viewed as lasting 8 s (*T*). Individual **A** exhibits veridical time perception (i.e., *P* = *T*; 1 subjective second = 1 s). Individual **B** would be viewed as having a *slower* internal clock (*P* > *T*), and individual **C** would be viewed as having a *faster* internal clock (*P* < *T*). The power function relating *P* to *T* (Glicksohn and Hadad, 2012) is given by: *P* = *aT*^β^, subsequently linearized as log(*P*) = log(*a*) + βlog(*T*) = α + βlog(*T*). For individual **A**, α = 0, and β = 1. When α ≠ 0, there is a consistent bias in producing durations; when β ≠ 1, then the untransformed data are not consistent with a linear function. We compute individual regressions of *P* on *T*, after log transformation, and thereby derive individual estimates for the two parameters, the intercept (α) and the slope (β) of this psychophysical function. In this study, as in previous studies (Glicksohn, 1992, 1996), we employ short target durations of 4, 8, 16, and 32 s. The TP task is not overly demanding, and is completed within less than 5 min. In previous reports employing a *Ganzfeld*, this task with these durations was found to reflect both the influence of the altered sensory environment (Glicksohn, 1992), and that of the participant’s personality interacting with such an environment (Glicksohn, 1996). In both cases, it is the *intercept* of the function which reveals both state and trait effects. In more recent work, we have shown how the same task also reveals the influence of trait mindfulness on time perception using experienced practitioners of mindfulness meditation (Berkovich-Ohana et al., 2012).

The current study improves on our earlier explorations in three notable ways. First, we employ a total whole-body immersive *Ganzfeld* coupled with both red and indigo-colored stimulation. The effect on time perception of exposure to such colored light has generated its own literature (Küller and Mikellides, 1983; Caldwell and Jones, 1985; Huang et al., 2012; Shibasaki and Masataka, 2014). We note that while Caldwell and Jones (1985) did not find a consistent effect of red versus blue light in their TP data, they do acknowledge that this might be due to the very short duration of exposure to such colored light (45 s). Indeed, exposure to colored light for such a short duration will not facilitate an immersive experience. In contrast, the Huang et al. (2012) study, allowing for exposure time in excess of 30 min to each colored light (red, blue, green), each on separate days, most certainly enabled an immersive experience. They reported a consistent shortening of produced duration when exposed to red colored light in their TP task, employing target durations of 180 s (they also employed a target duration of 600 s). To our mind, the use of such *lengthy* target durations is less informative than is our own use of different *short* target intervals, and this is on three counts. First, when employing lengthy target durations, in excess of what seems to be a maximal duration for time perception of around 100 s (Wackermann, 2007, p. 26), the very notion of time *perception* is compromised. Second, the use of a number of short target intervals, in contrast to one long target duration, enables the investigation of the psychophysical function for time perception, which is preferable to a focus on a single duration (Eisler, 1996, p. 67). Third, a lengthy target duration used for a TP task must surely be overtly disruptive of the effects of WBPD on the participant, because the participant is involved more in the TP task and less in the ongoing experience (Glicksohn, 2001a, p. 350). Given that in the present study, our participants are immersed in such colored light, for a period of 5 min for each color, we thereby enable better conditions for investigating such effects, on two counts. First, a period of 5 min exposure to such an altered sensory environment is ample time to enable an immersive experience (Hochberg et al., 1951, p. 155). Second, such exposure to each of two colored lights will enable us to investigate whether there is such a difference between the arousal potential of red and blue light (Caldwell and Jones, 1985).

Our second improvement on our earlier explorations is that instead of employing these four time intervals, each to be produced once (Glicksohn, 1992, 1996; Lipperman-Kreda and Glicksohn, 2006; Glicksohn and Hadad, 2012), we employ the same intervals, in two separate series. This is in line with other papers (Glicksohn et al., 2009; Berkovich-Ohana et al., 2012), hence providing us with additional data for assessing the hypothesized lengthening of TP.

Third, our participants are all experienced practitioners of breathing meditation. What we gain from this is the distinct possibility of obtaining a more informative phenomenological report of both subjective experience and of temporal experience. Such proficient meditators have been reported to be more introspectively accurate than are novices (Lutz et al., 2007), including regarding their bodily sensations (Fox et al., 2012), and to have an enhanced ability to sustain attention (Lutz et al., 2009). The downside of this prior extensive experience with meditation should also, however, be noted, and this is in two areas. First, while in the WBPD chamber, even if they are just “observing” or “resting,” they are also probably entering into their meditative mode (Tei et al., 2009, p. 163). While this is not at all detrimental to the goals of this study, which focuses on temporal experience, this should, nevertheless, be noted from the start. Second, and of more relevance to our working hypothesis, that exposure to – in fact, immersion in – WBPD should result in a lengthening of TP, is that such experienced practitioners of meditation should characteristically exhibit such a lengthening of TP (Kramer et al., 2013; Droit-Volet et al., 2015). Nevertheless, given that this is a within-subject design, we are looking at such a lengthening of produced duration post- relative to pre-exposure to WBPD.

## Materials and Methods

### Whole-Body Perceptual Deprivation (WBPD)

The WBPD chamber is in the shape of an egg (**Figure 1**), created by Patrizio Paoletti (Paoletti et al., 2017), and is located in the Cognitive Neurophysiology Laboratory, at the Research Institute for Neuroscience, Fondazione Patrizio Paoletti, Assisi, Italy. Two WBPD chambers were used. The first WBPD chamber had a diameter of 3 m and a height of 3.5 m, and opened and closed its top electronically. Following the translocation of the lab, a second WBPD chamber was utilized, having a diameter of 1.7 m and a height of 2.22 m; this chamber opens and closes manually (for security reasons, to avoid problems in case of an earthquake). In both chambers, the participants could sit comfortably inside upon a chair. Instructions were given verbally; sounds were transmitted via concealed speakers. The chamber was first flooded with white light, followed by red light and indigo light (these two colored-light conditions were presented in a counterbalanced order across participants), enabling a totally immersive WBPD. The participant’s verbal reports were heard through a microphone, and were recorded.

### Participants and General Procedure

The complete sample of this study comprised 32 participants, and included EEG recording (these EEG data will be presented elsewhere). All are experienced practitioners of breathing meditation, chosen to participate due to their enhanced introspective and reporting abilities. They were recruited from the *Ideas – Knowledge of Excellence, International School of Self-Awareness*^1^, and had been practicing breathing meditation from between 182 and 7280 h. They all completed a number of questionnaires prior to entering the WBPD chamber, which was illuminated with white light (5 min, eyes-closed condition). This was followed by red and indigo light, each presented for 5 min (eyes-open conditions). At the end of the session, the participants underwent an extensive interview. All subjects gave written informed consent in accordance with the Declaration of Helsinki. The protocol was approved by the ethics committee of Bar-Ilan University. Unfortunately, we have missing TP data for 13 participants, for the following reasons: (1) a malfunction of the chamber for one participant (S13); (2) a problem in recording post-WBPD TP for six participants (P2, P6, P8, P10, S10, and S11); and (3) a problem in recording TP, both pre- and post-WBPD, for six participants (P11, P13, P16, S1, S2, and S4). Hence, only 19 participants (10 males and 9 females, whose age ranged between 27 and 66 years) provided complete data (both TP and verbal report), and it is their data which are presented here.

### Time Production

Prior to entering the chamber, the participants completed a TP task. One group of 16 participants (S1–S16) then repeated the task within the chamber, at the end of the session; a second group of 16 participants (P1–P16) repeated the task after exiting the chamber.

Four short durations of 4, 8, 16, and 32 s served for this TP task. The participants were required to remain with eyes closed while producing each of these target durations, pressing a finger button when they estimated that the time that passed following a “beep” sound equaled the target duration. Each target interval was produced twice, the target durations being presented in two series, each having a random order of the four target durations. Produced (*P*) and target (*T*) durations were both log-transformed (to base 2), rendering thereby a linear scale for both ranging for *T* between 2 and 5, with a midpoint value of 3.5. Mean log(*P*) served as one dependent measure, having an expected value of 3.5. Log(*P*) was then regressed on log(*T*), providing for each participant two further dependent measures, an intercept value, and a slope value (**Table 1**), where the slope is equivalent to the exponent of the power function relating *P* to *T*.

**Table 1:** TP profile for each participant, both pre- and post-WBPD.

Participant		Staying in chamber (*n* = 10)									
		Pre					Post				
	Gender	Mean log(*P*)	Slope	Intercept	*F*(1,6)	*R*^2^	Mean log(*P*)	Slope	Intercept	*F*(1,6)	*R*^2^
S3	F	1.404	0.615^∗^	–0.750	118.94^∗^	0.952	1.363	0.808^∗^	–1.467	165.04^∗^	0.965
S5	M	3.978	0.996^∗^	0.493	65.68^∗^	0.916	4.263	0.997^∗^	0.773	124.28^∗^	0.954
S6	F	3.352	1.346	–1.357	18.15	0.752	3.447	1.131^∗^	–0.513	171.93^∗^	0.966
S7	F	3.284	0.879^∗^	0.209	545.40^∗^	0.989	3.489	1.150^∗^	–0.545	269.03^∗^	0.978
S8	M	3.323	1.143^∗^	–0.677	1501.26^∗^	0.996	3.193	1.122^∗^	–0.733	428.97^∗^	0.986
S9	M	3.420	1.086^∗^	–0.383	544.87^∗^	0.989	3.438	1.261^∗^	–0.977	129.45^∗^	0.956
S12	M	2.386	1.009^∗^	–1.147	55.65^∗^	0.903	2.391	1.188^∗^	–1.768	114.55^∗^	0.950
S14	F	3.526	1.078^∗^	–0.248	147.84^∗^	0.961	3.838	0.791^∗^	1.069	108.91^∗^	0.948
S15	M	2.372	0.950^∗^	–0.953	56.31^∗^	0.904	3.226	0.881^∗^	0.143	114.50^∗^	0.950
S16	F	2.814	0.946	–0.498	31.44	0.840	3.034	0.866^∗^	0.004	47.18^∗^	0.887
			**Exiting chamber (*n* = 9)**							
P1	M	3.613	1.283^∗^	–0.877	355.76^∗^	0.983	3.373	1.268^∗^	–1.067	666.38^∗^	0.991
P3	F	3.962	1.007^∗^	0.438	171.40^∗^	0.966	4.008	1.078^∗^	0.234	341.16^∗^	0.983
P4	F	3.589	1.021^∗^	0.014	2008.63^∗^	0.997	3.539	.970^∗^	0.143	1307.01^∗^	0.995
P5	F	3.600	1.004^∗^	0.084	1158.25^∗^	0.995	3.567	1.238^∗^	–0.765	47.43^∗^	0.888
P7	M	2.850	1.303^∗^	–1.710	545.37^∗^	0.989	2.726	1.298^∗^	–1.819	417.41^∗^	0.986
P9	M	3.604	1.185^∗^	–0.544	221.23^∗^	0.974	3.691	1.121^∗^	–0.233	425.43^∗^	0.986
P12	M	2.649	1.069^∗^	–1.094	179.44^∗^	0.968	3.344	1.167^∗^	–0.740	464.91^∗^	0.987
P14	M	3.796	1.137^∗^	–0.183	1095.00^∗^	0.995	3.657	1.195^∗^	–0.525	213.14^∗^	0.973
P15	F	3.514	1.209^∗^	–0.718	175.91^∗^	0.967	2.986	1.207^∗^	–1.240	641.84^∗^	0.991

*An asterisk indicates a significant (*p* < 0.05) effect.*

### The Semi-structured Interview

In the semi-structured interview, the participant was first asked to give a free description related to his/her experience. The participant was then asked to freely describe and also to rate on a one to nine scale (1 = low; 9 = high) his/her sense of: time, space, positive and negative emotions, bodily arousal, external and internal environment, metacognition and types of thoughts, concentration, insight, synesthesia, motor movements, sense of agency, and ownership inside the WBPD chamber. In addition, the participants were asked regarding thoughts about the past (memories), the future (concrete imagination), and momentary experiencing.

## Results

### Mean Log(*P*)

Mean log(*P*) ranged between 1.39 and 4.19 (*M* = 3.21, *SD* = 0.67) prior to exposure to the WBPD, and between 1.29 and 4.40 (*M* = 3.29, *SD* = 0.64) following exposure to the WBPD. As can be deduced from the minimum values reported above, one individual (S3) produced time intervals indicative of an extremely fast internal clock. For example, for a target duration of 32 s, she produced pre-WBPD durations of 6.1 and 5.0 s, and post-WBPD durations of 6.2 and 6.0 s. We shall analyze the data both including her, and when removing her, to see whether her data had a marked effect.

The first question to consider is whether there is an increase in mean log(*P*) from the first series to the second series of TP estimates, either pre- or post-WBPD. For the first set of data (pre-WBPD), for our group of 19 participants there is such an increase [*F*(1,18) = 6.31, MSE = 0.06, *p* < 0.05], with mean log(*P*) increasing from 3.11 (*SD* = 0.69) to 3.31 (*SD* = 0.66), thus tending to veridical time perception. Similar values hold when the outlying individual was removed. For the second set of data (post-WBPD), this trend should be analyzed taking into account the difference in experimental protocol. When this difference in protocol is entered as a grouping factor in a two-way ANOVA, the Group [TP post-WBPD assessed within the chamber (*n* = 10), or after exiting the chamber (*n* = 9)] × Series (first, second) interaction is significant [*F*(1,17) = 20.20, MSE = 0.02, *p* < 0.0005]. This interaction indicates that for those participants who remained within the chamber while performing TP, mean log(*P*) increased from 3.03 (*SD* = 0.79) in the first series to 3.31 (*SD* = 0.82) in the second; this replicates the same trend seen pre-WBPD. In contrast, for those participants who exited the chamber, mean log(*P*) decreased from 3.47 (*SD* = 0.38) to 3.39 (*SD* = 0.42). Again, similar values hold when the outlying individual was removed.

Thus, mean log(*P*) presents the following curvilinear trend when assessed four times, for those who remained within the chamber: 2.82, 3.16, 3.03, and 3.31. Note specifically the *decrease* in value from time 2 to time 3, in opposition to the hypothesized lengthening of TP as a function of exposure to WBPD. For the second group of participants, who exited the chamber, mean log(*P*) presents the following trend: 3.44, 3.49, 3.47, and 3.39. Note specifically the *stability* in value from time 2 to time 3, followed by a reduction in value at time 4.

For those nine participants exiting the chamber, a Time (pre-WBPD, post-WBPD) × Series two-way ANOVA revealed that neither Series nor Time (nor their interaction) were significant, indicating that if there was an effect for WBPD on TP, as noted above looking at all four data points, this was “washed out” quite quickly. In contrast, for those 10 participants who remained within the chamber, there was a main effect for Series [*F*(1,9) = 18.16, MSE = 0.05, *p* < 0.005], but no interaction of Series with Time. For Time there was a suggestive trend, which on removal of the outlying individual approached significance [*F*(1,8) = 4.82, MSE = 0.08, *p* = 0.059], whereby TP pre-WBPD (*M* = 3.16, *SD* = 0.59) *increased* post-WBPD (*M* = 3.37, *SD* = 0.53), in line with the hypothesis. Clearly, the inclusion of the outlying individual masks this trend.

The second question to consider is whether mean log(*P*) reveals a gender difference in TP, as has been previously reported (Glicksohn and Hadad, 2012). For the pre-WBPD data, the increase in mean log(*P*) from the first series to the second series of TP estimates is not moderated by Gender, neither is there a main effect for Gender, when employing a Gender × Series ANOVA. For the post-WBPD data, a Group × Gender × Series × Time ANOVA revealed no main effect and no interactions with Gender. Hence, gender is not a relevant factor in this study.

Our participants were first exposed to white light, followed by red and indigo light – with these two colored-light conditions being presented in a counterbalanced order across participants. Red light, compared to blue (indigo) light, has been argued to be more arousing, hence should speed up the internal clock (Glicksohn, 2001b), resulting in shorter TP (Huang et al., 2012). Hence, participants exposed to red-then-indigo illumination in the WBPD chamber should exhibit a lengthening of TP over time, while participants exposed to indigo-then-red illumination should exhibit comparatively shorter TP. One should also consider a potential gender difference here (Delay and Richardson, 1981; Shibasaki and Masataka, 2014). We ran a Color × Gender ANOVA on the difference score (post-WBPD – pre-WBPD), which uncovered a significant interaction [*F*(1,15) = 5.64, MSE = 0.07, *p* < 0.05], and no main effects. While our nine female participants showed practically no difference in TP score for either red-then-indigo illumination (*M* = 0.057, *SD* = 0.116, *n* = 4) and indigo-then-red illumination (*M* = -0.001, *SD* = 0.328, *n* = 5), our 10 male participants showed a marked increase in TP score for indigo-then-red illumination (*M* = 0.518, *SD* = 0.452, *n* = 3) compared to red-then-indigo illumination (*M* = -0.035, *SD* = 0.178, *n* = 7). An effect for color illumination only found for men has been previously reported (Shibasaki and Masataka, 2014); nevertheless, one would predict a *decrease* in TP score for indigo-then-red illumination, as noted above.

### Power Function

Log(*P*) was regressed on log(*T*), providing for each participant an intercept value and a slope value (**Table 1**). Given only four target durations, we consider an *r*^2^ value ≥ 0.95 as supporting linearity, as in previous publications (Lipperman-Kreda and Glicksohn, 2006; Glicksohn and Hadad, 2012). Inspection of both the individual log–log plots and the individual *r*^2^ values revealed that for pre-WBPD, the data of five individuals could not be considered to exhibit linearity following the log transformation. A similar inspection of the post-WBPD data revealed that for one of these individuals, this situation was continued. In addition, for two individuals, while their pre-WBPD data exhibited linearity following the log transformation, their post-WBPD data did not. We shall be analyzing the data for these seven individuals in separate.

**FIGURE 2:**
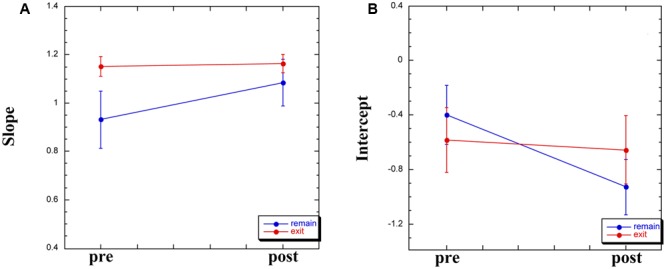
Slope **(A)** and intercept **(B)** measures derived from the log–log plot, pre- and post-WBPD.

For the remaining 12 participants, Group × Time (pre-WBPD, post-WBPD) ANOVAs were run on the slope and the intercept in separate. For the slope, there was both a main effect for Time [*F*(1,10) = 5.94, MSE = 0.04, *p* < 0.05] and also a Group × Time interaction [*F*(1,10) = 7.77, MSE = 0.04, *p* < 0.05]. As can be seen in **Figure 2A**, for those participants exiting the chamber there is practically no increase in slope due to WBPD, while for those participants who remained within the chamber, their pre-WBPD slope is, surprisingly, markedly lower than that of the other slope values. We have no ready explanation for this, and thus suggest treating the slope measure in this study with caution.

Turning to the intercept, we found both a main effect for Time [*F*(1,10) = 6.09, MSE = 0.05, *p* < 0.05] and a Group × Time interaction [*F*(1,10) = 5.63, MSE = 0.05, *p* < 0.05]. As can be seen in **Figure 2B**, for those participants exiting the chamber there is practically no decrease in intercept due to WBPD, while for those participants who remained within the chamber, their post-WBPD intercept is markedly *lower*. An *increase* in mean log(*P*) post-WBPD, even if only appearing as a trend in the data (see above), coupled with a *lower* intercept suggests an interaction between Time and Duration. In the next analysis, we looked at the individual log–log plots, both pre- and post-WBPD.

### Variability in Individual Power Functions

**FIGURE 3:**
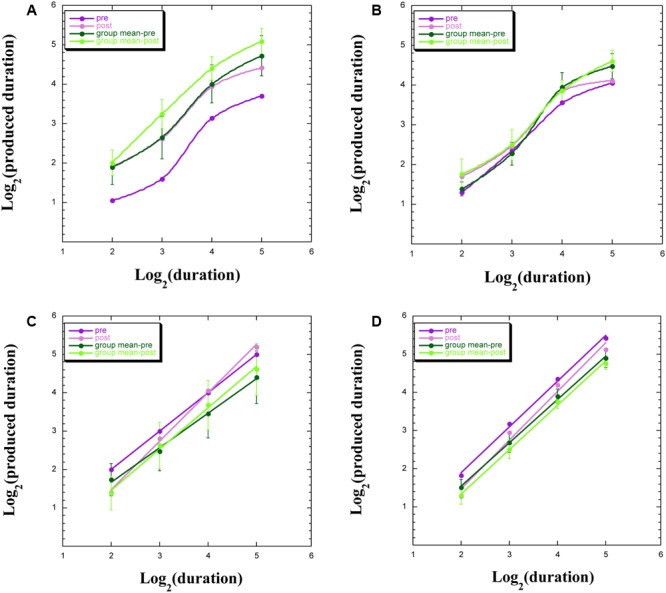
Produced duration as a function of target duration, both after log transformation for: **(A)** individuals not exhibiting linearity; **(B)** individuals not exhibiting a clear distinction between pre- and post-WBPD TP data; **(C)** individuals exhibiting a change in both slope and intercept from pre- to post-WBPD; **(D)** individuals exhibiting parallel functions for pre- and for post-WBPD.

We uncovered four common profiles within our data, both for participants remaining within the chamber and for those exiting the chamber, with exemplars appearing in **Figure 3**. These four profiles indicate a discontinuity in psychophysical function (**Figure 3A**), signifying aberrant TP; an overlap of functions pre-WBPD and post-WBPD (**Figure 3B**), suggesting no clear influence of WBPD; longer productions post-WBPD, in line with our hypothesis (**Figure 3C**); and shorter productions post-WBPD, in contrast to our hypothesis (**Figure 3D**).

**Figure 3A** exemplifies the data of S15, who is one of three (S15, S5, and P5) of the seven individuals not exhibiting linearity following the log transformation (two of these three had remained within the chamber), together with the group means [±standard error (SE)], which appear in the upper curves. For all four curves appearing in this panel, we have used the “smooth curve” fit provided by KaleidaGraph software. What one notes here is that in spite of this lack of linearity, the post-WBPD data exhibit *longer* productions than the pre-WBPD data, in line with the hypothesis. An intriguing possibility is suggested by these data: Linearity for the first two data points, and linearity for the last two data points, with discontinuity of the function between these regions. The difference between these four linear functions would be primarily revealed in the intercepts.

**Figure 3B** exemplifies the data of S16, who is one of the remaining four (S16, S12, S14, and S6) of the seven individuals not exhibiting linearity following the log transformation (all four of whom had remained within the chamber). We also present the group means (±SE). What one notes here is that pre-WBPD and post-WBPD data are intertwined – with no clear dominant trend in the data.

**Figure 3C** exemplifies the data of S7, who is one of four (S7, S9, S3, and P3) individuals (three of these had remained within the chamber), for whom the post-WBPD function diverges from the pre-WBPD function primarily for the larger target durations, indicating *longer* productions post-WBPD, in line with the hypothesis. Their data are suggestive of the Duration × Time interaction noted above, wherein both intercept and slope change from pre- to post-WBPD. We also present the group means (±SE), supporting such an interaction. For both individual data and for group data, a linear fit in this log–log plot is clearly seen.

**Figure 3D** exemplifies the data for P1, who is one of six (P1, P7, P14, S8, P4, and P15) individuals (five of these had exited the chamber), for whom the post-WBPD data exhibit uniformly *shorter* productions, in opposition to the hypothesis. We also present the group means (±SE). The reverse pattern, whereby post-WBPD data exhibit uniformly longer productions, in line with the hypothesis, is found for the remaining two (P9 and P12) individuals, both of whom had also exited the chamber. For both individual data and for group data, a linear fit in this log–log plot is clearly seen.

### The Experience of Time

A question is raised as to the extent to which this variability in our TP data is related to variability in the experience of time while immersed in WBPD. We block our experiential data in line with the four major groups appearing in **Figure 3**.

Two participants whose TP data are exemplified by **Figure 3A**, who had remained within the chamber, reported the following in the interview at the end of the session: “There was no focus on [time]. The time dimension lost its meaning and significance” (S5); “It felt as if more time had passed. Time was expanded, I perceived more the passage of time; time passes” (S15). These reports do not indicate any apparent discontinuity in time perception, and in fact seem to be dissociated from TP performance.

Three other participants, who had also remained within the chamber, whose data are exemplified by **Figure 3B**, reported the following in the interview: “[Time] disappeared” (S12); “Slow, longer” (S14); and “No time” (S16). If the sensation of time “disappeared,” then perhaps their TP performance should be haphazard, as seen in **Figure 3B**.

Three participants who had also remained within the chamber, whose data are exemplified by the type of interaction appearing in **Figure 3C**, reported the following: “Time was slower” (S7); “Expanded, flowing” (S9); and “The cognition of time after the lights, I didn’t know what would have happened” (S3). If “time was slower,” then one would expect to see this experience reflected in the TP data by longer productions, especially for longer target intervals, as can be seen in **Figure 3C**.

The majority of the participants presenting with TP data in opposition to the hypothesis of there being a lengthening of produced time had exited the chamber. The reports of three of these, whose data are exemplified by **Figure 3D**, are as follows: “Concerning time, time was not fast or slow, but I was firm with myself. There was absence of time, but I was setting my own time, for example with my breath, or my sensations, thus it was set on the present, it was just what it was” (P15); “It seemed that there was no time. I didn’t think about it” (P7); and “Inside there I didn’t have time perception, however when I did the exercise afterward, I realized that I had a more refined time perception … I didn’t perceive time, it was expanded” (P4). It could well be that on exiting the chamber, post-WBPD TP performance became dissociated from the timelessness that these participants reported regarding their WBPD experience. Two other participants, who had also exited the chamber, present the reverse pattern to that seen in **Figure 3D**, in line with the hypothesis. This is what they reported: “Very expanded. I had the feeling to be in a not defined space, and also time was not defined. However, practical thought about what time it is, what I have to do later, set in” (P9); “It was very slow” (P12). If time was “very expanded” and/or “very slow,” then one would expect to see this reflected in the TP task by longer productions, which was the case for these participants (even though they had exited the chamber).

## Discussion

In the current study, we examined the effects of a total whole-body immersive *Ganzfeld* coupled with both red and indigo-colored stimulation on TP and temporal experience. Clearly, as Block (1979, p. 202) suggests, “it is quite reasonable to determine whether gross reductions in external stimulus information affect temporal experience.” We are, of course, in full agreement with Morrison and Hunt (1996, p. 118) regarding the need “to turn to the content-analyzed interview when assessing subjective experience.” In doing so, we find wide individual differences in both temporal experience and TP among participants immersed in our WBPD environment.

Our working hypothesis was that exposure to – in fact, immersion in – WBPD should result in a lengthening of TP. Our study was designed such that we could maximize this effect, for our participants were all experienced meditators (in the widest sense of the term). Such a population should exhibit a lengthening of TP during meditation (Glicksohn, 2001b), and also exhibit longer TP while not meditating, at baseline (Berkovich-Ohana et al., 2012). Consider the following: The *Ganzfeld* (and other conditions of restricted environmental stimulation) comprises “… an externally structured analog of meditation and similar states” (Suedfeld, 1980, p. 44); and the effects of a Turrell *Ganzfeld* have been “… frequently described as calming, relaxing, womb-like, uplifting, meditative and so on” (Benson, 2001, p. 125). Conversely, “certain meditative practices … have perceptual and cognitive outcomes similar to sensory deprivation” (Lindahl et al., 2014). Hence, we have compatibility between trait and state in expecting such a lengthening of TP.

Not all our participants exhibit the hypothesized lengthening effect; some, in fact, exhibited shorter TP following WBPD. We find that for those participants whose data exhibited linearity in the log–log plot of produced duration to target duration, it was the *intercept* of this function which was the locus of the effect for WBPD, much as was reported in a previous study (Glicksohn, 1996) employing both altered sensory environments (including *Ganzfeld*) and TP (using the same target durations). While an *increase* in the intercept might be due to the repetition of the task, and not necessarily due to exposure to an altered sensory environment (Glicksohn, 1996, p. 368), here we note a marked *decrease* in intercept due to exposure to WBPD, for those participants who remained in the chamber. For those exiting the chamber, on the other hand, there was practically no decrease in intercept due to WBPD.

We also noted that for a number of participants not exhibiting such linearity in their data, the difference between the two functions describing their data would be revealed in the intercept. For them, their post-WBPD intercept is *higher*. It might well be that we have uncovered the same type of “break” in the psychophysical function, emphasized by Eisler (1990) using the method of reproduction. In our data, we noted such a discontinuity in function between 4 and 8 s – something never observed before using our TP task (but also never actively investigated before). A discontinuity in function above 4 s, while not quite conforming to the hypothesized 3-s “subjective” or “sensible” present (Pöppel, 1997; Wackermann, 2007), would nevertheless conform to the temporal location of the break observed by Eisler (1996). As Eisler (1996, p. 77) writes: “For durations below about 4 s on the average there seems to be no difference between male and female subjects. Longer durations, above the break, yield longer reproductions for female subjects.” Furthermore, as Eisler et al. (2004, p. 265) have indicated, “… for almost all subjects the psychophysical function showed a break or discontinuity at different temporal locations for different individuals … .” The explanation suggested for finding the locus of the effect in the intercept of the function was that the intercept reflects some “bias or error in production” (Glicksohn, 1996, p. 367). Such an error aligns with the claim that the internal clock incorporates a “fallible” counter (Killeen and Taylor, 2000), which would further predict such discontinuities in the psychophysical function, as seen here for these three individuals.

In spite of the existence of these individual differences – or, better, because of these individual differences – we can make the following tentative claims. First, when “time disappeared,” TP becomes haphazard. Second, when “time was slower” or “time was expanded,” TP is lengthened. We have also learned that the effects of WBPD are not long-lasting: Participants who remained in the chamber tended to report time as being slower, and tended to exhibit a lengthening of TP, as hypothesized; participants who exited the chamber tended to exhibit shorter productions, in opposition to the hypothesis. One might question whether it would have been better to ask our participants to produce durations during WBPD, rather than following WBPD. One could argue either way: If TP reflects time-in-passing (Glicksohn, 2001b), then performing TP during WBPD would be more tightly related to temporal experience during WBPD. TP can, in fact, serve as a measure of mental workload (Zakay et al., 1999, pp. 568–570; Baldauf et al., 2009), and will fluctuate as one’s level of vigilance changes – but that is exactly what should be happening in the *Ganzfeld* (Avant, 1965).

On the other hand, by performing TP during WBPD, using a task employing the production of multiple target durations, this might very well disrupt one’s temporal experience, and one’s subjective experience in general, which is of prime interest for studies of WBPD. In fact, any task might disrupt the effects of WBPD (Suedfeld, 1980, pp. 67–68; Glicksohn, 2001a, p. 350). Thus, TP following WBPD is not necessarily a limitation of the present study. This, however, is an issue worth considering in future studies in this domain.

Another point to consider is the fact that, as part of the structured interview, we had requested our participants to rate on a one to nine scale (1 = low; 9 = high) their sense of time. For the majority of our participants (*n* = 18), this was a nonsensical idea, and they could not make such a rating. Two gave a rating of “0” (not on the scale), one gave the verbal rating of “neutral,” another gave a verbal rating of “medium,” and the other participants gave a numerical rating. The astonishment at the very question expressed by a number of our participants brings to mind a comment made by Klüver (1926, p. 512): “A question of the experimenter concerning time was considered rather ridiculous. It seemed to me incommensurate to speak about the experienced abundance of phenomena in terms of minutes and hours.”

A reviewer of this paper has astutely commented that in employing two different WBPD chambers, we might have impacted on TP, because the second chamber was smaller in size relative to the first. We further note that one group of 16 participants (S1–S16) repeated the TP task within the larger chamber, at the end of the session, while the second group of 16 participants (P1–P16) repeated the task after exiting the smaller chamber. There is a scanty literature that bears on the possible effect of the WBPD chamber size on temporal experience. DeLong (1981) found that observers of differently scaled environments (e.g., one-sixth of the full size of a room), who were asked to imagine themselves as being embedded within the scaled-model, exhibited a TP of a target duration of 30 min that was proportional to the scale of the environment. More recently, Zäch and Brugger (2008) reported a study wherein their participants were asked to imagine, with eyes closed, a railway clock at either a close (30 cm) or a far (6 m) distance from them, focusing on the imagined movement of the clock’s second hand, TP of 15 and 30 s was *shorter* for the clock imagined to be *farther* away. If chamber size did have an impact in this study, one would assume that TP would be shorter in the larger chamber. However, as we have stressed above, individual differences in both TP and temporal experience seem to predominate over other design factors in this study.

Finally, what bearing does the present study have for the study of time perception and clinical disorders? One can view this study in analogy to another research domain, presenting with similar reports of such aberrant temporal experience – depression. Consider the reports of some of our participants, indicating for them that “time was slower,” that “time was expanded,” or that “time disappeared.” Would such reports be readily distinguished from reports made by depressed individuals that “… time seems to pass more slowly than usual or even stops” (Droit-Volet, 2013, p. 260), as also emphasized by other researchers looking at time perception and depression (Oberfeld et al., 2014, p. 1)? If “time was slower,” then one would expect to see this experience reflected in the TP data by longer productions, especially for longer target intervals, as can be seen in the present study (**Figure 3C**). One would conclude that the internal clock in both the present *Ganzfeld* setup and in depression had slowed down (Glicksohn, 2001b). Indeed, this would be the same inference to be drawn regarding meditation (Glicksohn, 2001b; Wittmann and Schmidt, 2014; Wittmann et al., 2015).

And yet the study of time perception in depression is plagued by inconsistency, at a number of levels. Oberfeld et al. (2014, p. 4) assume that if “… altered time perception in depressive patients in terms of a faster running internal clock is true, we expect the subjects in the depressive group to underproduce … time intervals in the time production task… .” In contrast, we would argue for a *slower* internal clock, hence for *longer* TP. We make this claim, irrespective of actual findings in the literature, which are mixed (Droit-Volet, 2013; Thönes and Oberfeld, 2015; Mioni et al., 2016).

If the results of the present study do have relevance for the literature on time perception in depression, this would be in two ways. First, the locus of the effect should be found in the intercept of the psychophysical function, when produced time is plotted as a function of target duration, using a log–log plot, as in the present study. Preliminary support for this is clearly seen in the study reported by Kornbrot et al. (2013). Second, there are individual differences in both TP and temporal experience – and hopefully an analysis of both in studies of depression will move that line of research forward. We can conclude in full support of Droit-Volet (2013, p. 260), writing about time perception and depression, but with clear import for our own study: “It is therefore important to distinguish between the explicit awareness of time and the direct perception of time. A disturbance in the former does not systematically imply a disturbance in the latter. That said, time awareness may sometimes affect time judgments to a certain extent.”

## Author Contributions

JG, AB-O, and TB-S sponsored the study and wrote the manuscript. AB-O, TB-S, and FM ran the study.

## Conflict of Interest Statement

The authors declare that the research was conducted in the absence of any commercial or financial relationships that could be construed as a potential conflict of interest. The reviewer MP declared a shared affiliation, though no other collaboration, with one of the authors FM to the handling Editor.

## Footnotes

1 http://schoolofselfawareness.org/index.php/pages/page/40
